# Sci-tech analysis and related research on three glass beads unearthed from M686 in the Qin cemetery of Warring States Period in Hejia, Zhouling

**DOI:** 10.1371/journal.pone.0331048

**Published:** 2025-08-29

**Authors:** Wenhui Ha, Feng Sun, Congwen Zhai

**Affiliations:** China-Central Asia “Belt and Road” Joint Laboratory on Human and Environment Research, Key Laboratory of Cultural Heritage Research and Conservation, School of Cultural Heritage, Northwest University, Xi’an, China; National Research Centre (NRC), EGYPT

## Abstract

This study presents a systematic analysis of three glass beads excavated from Tomb M686 in the Qin Cemetery of Warring States Period (475 BC-207 BC) in Hejia,Zhouling. The aim is to reveal their material characteristics and weathering mechanisms. Using super-depth-of-field 3D video microscopy system (OM), X-ray fluorescence spectroscopy (XRF), and micro-Raman spectroscopy (Raman), all three glass beads were identified as the unique lead-barium silicate glass system of ancient China. The primary coloring element in the glass is Cu(II), and the main weathering products include PbCO₃ and BaSO₄. Focusing mainly on the cross-section of Sample 2, scanning electron microscopy with energy dispersive spectroscopy (SEM-EDS) and X-ray photoelectron spectroscopy (XPS) were employed to investigate the microstructural and compositional changes. The results revealed continuous leaching of Pb, Ba, and S elements from the glass substrate during the weathering process, interacting with substances in the burial environment to form stable weathering products PbCO₃ and BaSO₄. Based on the analysis of elements in the buried environment soil, indicated that barium has higher water solubility than lead. The weathering process was also accompanied by the conversion of Cu(Ⅱ) to Cu(Ⅰ), which explains the color alteration associated with glass weathering. This research is the first to integrate multiple microscopic techniques to systematically explore the weathering processes of lead-barium glass from the Warring States period, elucidating both its coloring mechanism and the formation pathways of weathering minerals. The findings provide technical support and a theoretical foundation for the understanding and preservation of related cultural relics.

## Introduction

Ancient Chinese glass holds unique value due to the diversity of its composition and manufacturing techniques. Based on chemical composition, it can be broadly classified into potash-lime glass, lead-barium glass, and potash glass. Among these, lead-barium glass is a unique glass system found only in China. It first appeared in the early Warring States period (475 BC-207 BC) and continued into the Eastern Han Dynasty (AD 25-AD 220), with its distribution spreading from the Central Plains to surrounding regions [1]. This type of glass is characterized by its high lead and barium content and differs significantly in production techniques from those used in other parts of the world during the same period, reflecting distinct regional and technological independence. With the large-scale excavation of lead-barium glass, scientists have employed various technical methods to study aspects such as its manufacturing techniques [2], raw material sources [3], and weathering mechanisms [4,5]. Commonly used analytical methods include X-ray fluorescence (XRF), laser ablation inductively coupled plasma atomic emission spectroscopy (LA-ICP-AES), and Raman spectroscopy. These are primarily used to identify weathering products such as lead carbonate (PbCO₃), barium sulfate (BaSO₄), barium silicate (BaSiO₃), and lead silicate (PbSiO₃). However, most current studies remain focused on the analysis of surface products, making it difficult to reconstruct the complete weathering process or assess the influence of burial environments on material degradation.

In recent years, some studies have attempted to overcome these limitations. For example, in 2023, Wang Yingzhu conducted a study on the corrosion layers of Qin Dynasty (221 BC-207 BC) lead-barium glass using optical microscopy, scanning electron microscopy, and laser Raman spectroscopy, revealing more significant barium leaching and suggesting that the weathering behavior was influenced by fluctuations in the pH of the burial environment [4]. In 2024, Li Jingyu used multimodal non-destructive techniques to analyze “dragonfly eye” glass beads and not only confirmed common weathering phases but also, for the first time, identified a rare blue weathering product: barium phosphate chloride [Ba₅(PO₄)₃Cl] [6]. These studies further highlight the complexity of the weathering process, which is governed by both intrinsic material properties and multiple environmental factors.

Meanwhile, research in materials science has demonstrated that compositional modification can significantly improve the structure, optical properties, and thermal stability of modern silicate and phosphate glasses. Advances in functional glass materials provide valuable theoretical insights into the weathering mechanisms of ancient lead-barium glass. Despite differences in application contexts, there are strong parallels in terms of compositional tuning, structural evolution, and performance response.For example, doping TiO₂ into the glass substrate has been shown to enhance network connectivity and thermal stability [7,8]. The incorporation of MoO₃ and La₂O₃ can improve the elastic modulus, density, and radiation shielding properties of glass [9,10]. Additionally, the manipulation of nanostructures and ionic valence states can synergistically enhance thermal, electrical, and magnetic properties, suggesting that changes in the valence states of metal ions during weathering not only affect color but also influence the overall material stability [11,12].These studies highlight the critical role of ionic behavior and microstructural features in the long-term stability of glass, offering valuable implications for the analysis of archaeological glass as well.

However, current studies on the weathering mechanisms of archaeological glass often rely solely on the analysis of surface weathering products to infer reaction pathways, lacking comprehensive investigations that link the burial environment with the internal structure of the material. Since the weathering process is typically influenced by multiple factors—such as pH, redox potential, moisture, and soil chemistry—surface characterization alone is insufficient to fully reveal the deterioration mechanisms. Therefore, it is urgently necessary to adopt an integrated research approach that includes burial soil analysis, cross-sectional structural and compositional analysis, and valence state determination. In this context, X-ray photoelectron spectroscopy (XPS) plays an irreplaceable role in revealing elemental valence states and redox behavior.

This study focuses on three glass beads with different degrees of weathering, unearthed from tomb M686 in the Qin Cemetery of Warring States Period (475 BC-207 BC) in Hejia,Zhouling. A comprehensive preliminary scientific analysis was conducted using a super-depth microscope (SDM), X-ray fluorescence spectroscopy (XRF), and micro-laser Raman spectroscopy was employed to analyze surface morphology and weathering products.. For Sample 2, cross-sectional analysis was conducted using scanning electron microscopy with energy-dispersive spectroscopy (SEM-EDS), enabling both line and area scans from the weathered surface to the interior substrate. This provided a three-dimensional characterization of elemental distribution resulting from glass weathering. Additionally, X-ray photoelectron spectroscopy (XPS) was used to interpret the nature of color changes in the glass from the perspective of elemental valence state transitions. By integrating internal structural analysis with consideration of the burial environment, this study offers a systematic analytical approach and a new perspective for the investigation of ancient glass weathering and preservation.

## Materials

**Fig 1:**
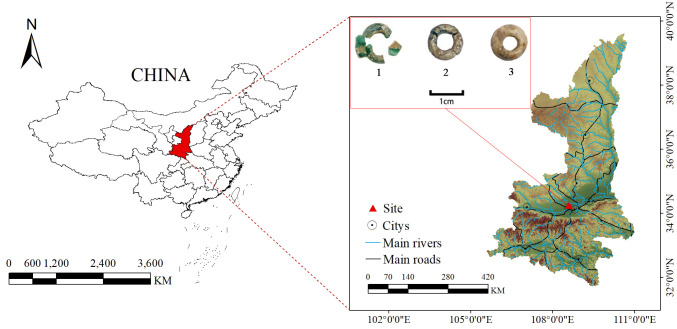
Location and sample diagram of the Qin tomb of Zhou Ling Hejia in the Warring States Period.

Qin Cemetery of Warring States Period in Hejia,Zhouling is located in the former site of Hejia Village, Zhouling Town, Weicheng District, Xianyang, Shaanxi Province. It is on the second-level platform on the north bank of the Wei River, 10.4 kilometers to the east from the site of Qin Xianyang City, and 1.7 kilometers to the northwest is the cemetery of King Wu of Zhou, as shown in Fig 1. Since August 2020, to support the infrastructure development of the Xi’an-Xianyang New City in the Western Xi’an New Area, the Shaanxi Provincial Institute of Archaeology has conducted ongoing excavations at the Hejia cemetery, uncovering a large number of burial sites, ditches, and road remains. To date, more than 1,400 graves have been excavated, The excavation unearthed a total of over 2,800 pieces (groups) of burial objects, including daily utensils, weapons, etc. Among which pottery is the bulk. Copper objects mainly are copper belt hooks. Additionally, a significant number of silicate artifacts, including a large number of beads. The objects of this study are three glass beads unearthed from M686, which show clear signs of weathering, as shown in the inset of Fig 1.

The three glass beads are all ring-shaped, with a green substrate, and their surfaces are covered with weathering products. Due to varying preservation conditions, the samples are numbered from 1 to 3 in order of increasing weathering severity.

Sample 1: Broken into four pieces, with lighter weathering and not fully covered by weathering products. The substrate color is still visible through the surface, and the weathering products, mainly yellow and white, are unevenly distributed on the surface of the green transparent glass. The bead has a hole diameter of 4.4 mm, a maximum diameter of 8.8 mm, and a thickness of 2.8 mm.

Sample 2: Broken into three pieces, with a mixed yellow and white weathering crust more widespread and thicker than in Sample 1. The condition of the glass substrate inside is difficult to discern from the surface. The hole diameter is 5.7 mm, the maximum diameter is 9.2 mm, and the thickness is 3.0 mm.

Sample 3: Well-preserved, with a yellow and white mixed weathering layer evenly enveloping the glass substrate surface. Compared to Samples 1 and 2, this sample shows the most severe weathering, with the green glass substrate only visible through the surface weathering products. The hole diameter is 4.0 mm, the maximum diameter is 8.9 mm, and the thickness is 6.4 mm.

## Methods

### OM analysis

The microstructure of the glass beads was observed using a high-depth-of-field 3D video microscopy system. The instrument model used was KH-7700, manufactured by Hoshi, Japan. The experimental conditions included the use of a halogen cold light source, along with various lenses and adapters, to observe the samples at magnifications ranging from 50–400 × . The study utilized the high-depth-of-field 3D video microscopy system to examine the surface details of both the glass substrate and the weathered layer.

### XRF analysis

The ARTAX-400 mobile X-ray fluorescence spectrometer of Bruker, Germany, measured conditions: voltage 30kv, current 900 μA, atmosphere Helium, determination time 300s; The standard curve is corning glass BCD.

Elemental analysis tests were carried out at random at three to five points on the substrate and weathered layer of the three glass samples, and the average value of the measured results at each part was taken as the final element content result to explore the elemental composition of the samples.

### SEM-EDS analysis

VEGA3XM tungsten filament scanning electron microscope produced by TESCAN, Czech Republic, was used to analyze, combined with X-ACT X-ray spectrometer produced by OXFORD, UK, for the observation of glass bead substrate and weathering and other surfaces, as well as the qualitative and quantitative analysis of elements. Test conditions: voltage 10–20 kV, magnification 100–1000×, working distance (WD) 13.08–14.37 mm, BI intensity 8–15. Three to five regions of each sample were averaged.

Scanning electron microscopy was used to observe the scattering and backscattering of substrate and weathered layer of sample 2, and the element surface distribution analysis and line scanning analysis were carried out to observe the presence of different elements in the sample.

### Raman analysis

DXR 2 microscopic laser Raman spectrometer produced by Thermo Field Company of the United States, equipped with 633 nm ion laser, acquisition spectrum range of 50 ~ 3500 cm^-1^, microscope eyepiece magnification of 10×, objective magnification of 50×, can be spatially resolved in situ nondestructive testing. The substrate and weathered layer of glass samples 1–3 were analyzed by Raman spectrometer, and the results of each phase of the samples were obtained.

### XPS analysis

The PHI-5000 VersaProbe Ⅲ type X-ray photoelectron spectrometer produced by ULVAC-PHI Company was used to measure the sample. A1K anode, X-ray beam spot size 650 spot scale, full spectrum scanning step size 1.000eV, fine spectrum scanning step size 0.050eV, full transmission scanning energy 20.000eV, Charge correction is performed at 1s (284.8eV) of carbon. This instrument is used to detect the valence state of Cu element in sample substrate and weathered layer, and discuss the weathering behavior of Cu element during the weathering process of lead-barium glass.

## Results

### Micromorphology

**Fig 2:**
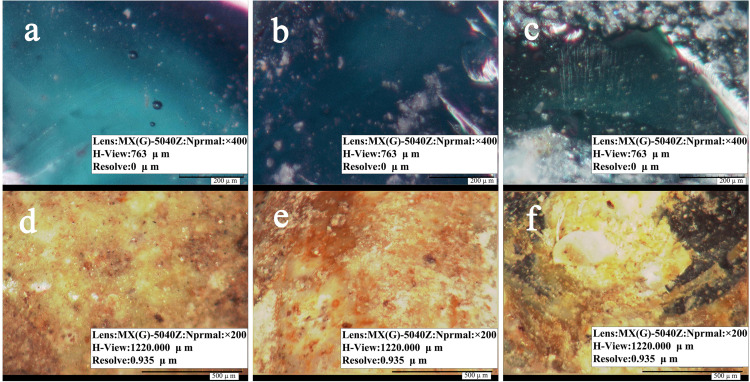
Micrograph of glass beads substrate and weathered layer.

Fig 2 (a-c) is a micrograph of three green glass beads magnified 400 × . The glass substrate is green and clear, and there are bubbles generated during the melting process. The weathering degree of the three glass beads substrate intensified in turn, manifested as white spot-like to clumpy cotton, and the weathering parts became cloudy.

Fig 2(d-f) is a 200 × magnification micrograph of the weathered layer on the surface of the three glass beads. The weathered layer mainly exists in a mixture of yellow and white colors, which is relatively smooth and has fewer pores. The green part of sample 3 is the glass substrate that is not completely weathered, and both the external exposed glass substrate and weathered layer exist in opaque state.

### Elemental composition

**Fig 3:**
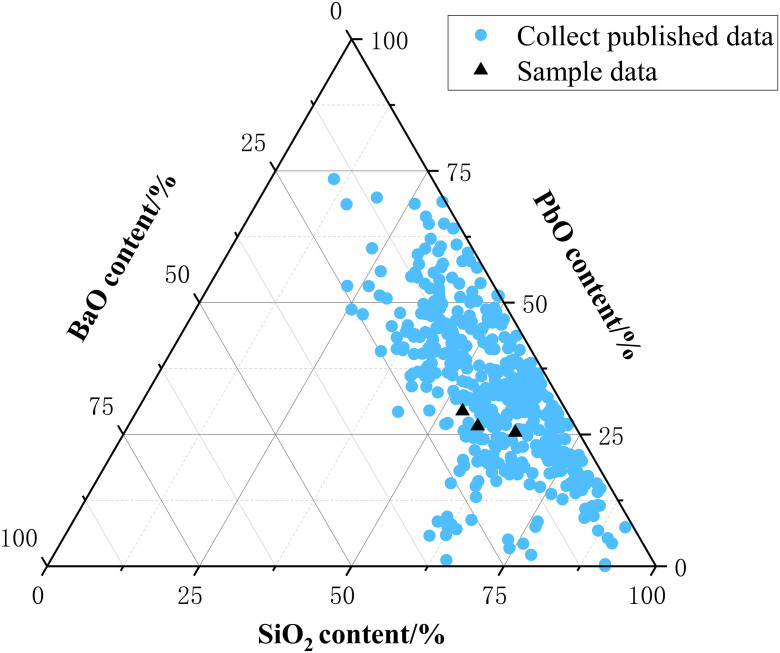
Scatter plot of distribution of main elements in lead-barium glass.

Based on the quantitative analysis of elements by X-ray fluorescence spectra of three samples, as shown in Table 1, the main components of the substrate of the samples are the same as those in the weathered layer. The content of PbO, BaO and SiO_2_ in the substrate ranges from 21.44% to 27.24%, 8.80% to 15.79% and 49.57% to 54.25%, respectively. The published content of main elements in lead-barium glass was collected [2,13–16], and compared with the sample data in this study, as shown in Fig 3. The samples in this study conform to the characteristics of the elemental composition of lead-barium glass, so all the three glass beads belong to the lead-barium silicate glass system. The content of Pb element in substrate is less than that in weathered layer, the weathering phenomenon may be accompanied by the outward migration and accumulation of Pb element. The content of Ba element in the substrate of sample 1 and sample 3 are more than that in the weathered layer, while sample 2 is the opposite, which means that the distribution of Ba element on the weathered layer surface of glass beads is not homogeneous, and there are barium-rich regions in the weathered layer of glass beads. The uneven distribution of barium element on the surface is due to the good water solubility of barium salts [4]. The Si element contents in the substrates are all more than those in the weathered layers, indicating that the weathering process destroyed the glass structure of the substrate, leading to the loss of Si element. The weathered layer contains a large amount of P element, which is presumed to be the effect of bones in the burial environment on it. K_2_O content ranges from 0.02% to 0.13%, Na_2_O content ranges from 1.72% to 5.23%, MgO content ranges from 0.05% to 1.55%, and CaO content ranges from 0.95% to 2.47%, which means that the flux for melting the glass is most likely to be plant ash. The coloration of ancient glass is not only related to the acidity or alkalinity of the chemical composition of the glass, but also relies on transition metal elements, such as iron, manganese, cobalt, nickel, chromium, and copper [17–21], which are usually present in the glass in the state of metastable ions, and their valence state is related to the melting atmosphere of glass (oxidation or reduction) [22]. XRF tests show that all three glass beads contain three elements, Fe, Mn, and Cu. Due to the low molar absorption coefficients of Fe and Mn ions, manganese is colorless when combined with iron in glass [23] and the low elemental content, the main chromogenic element of the three glass beads in this study should be Cu.

**Table 1:** Quantitative analysis results of X-ray fluorescence spectra of glass beads samples (wt%).

Sample	Substrate			Weathered layer		
	1	2	3	1	2	3
Na_2_O	2.76	5.23	1.72	2.73	0.59	1.26
MgO	0.15	1.55	0.05	0.56	0.47	0.26
Al_2_O_3_	0.67	5.01	1.20	1.19	0.78	1.04
SiO_2_	54.03	54.25	49.57	12.71	24.22	15.16
P_2_O_5_	0.79	0.99	0.56	39.55	17.77	35.12
K_2_O	0.00	0.13	0.02	0.03	0.51	0.04
CaO	0.95	1.34	2.47	3.08	5.98	4.17
TiO_2_	0.00	0.55	0.76	0.08	0.65	0.01
MnO	0.04	0.06	0.03	0.02	0.03	0.02
Fe_2_O_3_	0.12	0.29	0.11	0.27	2.01	0.17
CuO	0.39	0.36	0.47	0.30	3.27	0.33
BaO	15.05	8.80	15.79	9.52	15.22	8.91
PbO	25.03	21.44	27.24	29.98	28.51	33.52

### Physical phase structure

The Raman spectra of all three glass beads substrate have envelopes near 500 cm^-1^ and 1000 cm^-1^, which correspond to the symmetric stretching vibration of Si-O and the anti-symmetric stretching vibration of Si-O [24], proving that they are indeed glass. Raman analysis of the surface weathering products of the three samples was carried out separately, and the white and yellow weathering results of the three samples were similar, with peaks at 1053.69 cm^-1^ and 985.29 cm^-1^, corresponding to the phases of PbCO_3_ and BaSO_4_.

### Weathering behavior of lead-barium glass

**Fig 4:**
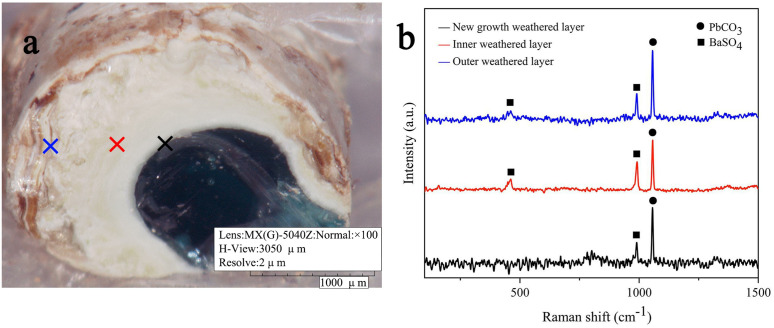
Photo and Raman spectrum of sample 2 cross-section.

The study of the weathering behavior of lead-barium glass mainly focuses on its morphology and elemental migration after weathering. The weathering degree of sample 2 is in the middle, and one of the fragments was selected. The green glass substrate and obviously different inner and outer weathered layers can be observed in its cross section, as shown in Fig 4a. Therefore, the cross section of sample 2 was selected as the experimental sample for the study of weathering behavior of lead-barium glass.

Weathering test points of different degrees were selected on the cross section of sample 2 for Raman spectral analysis, and the results are shown in Fig 4b. There are PbCO_3_ and BaSO_4_ in both inner and outer weathered layer, and the content of BaSO_4_ in new growth weathered layer near the substrate is the least, the content of BaSO_4_ in inner weathered layer is increased, the content of BaSO_4_ in outer weathered layer is slightly decreased. The overall weathering products were mainly PbCO_3_.

**Fig 5:**
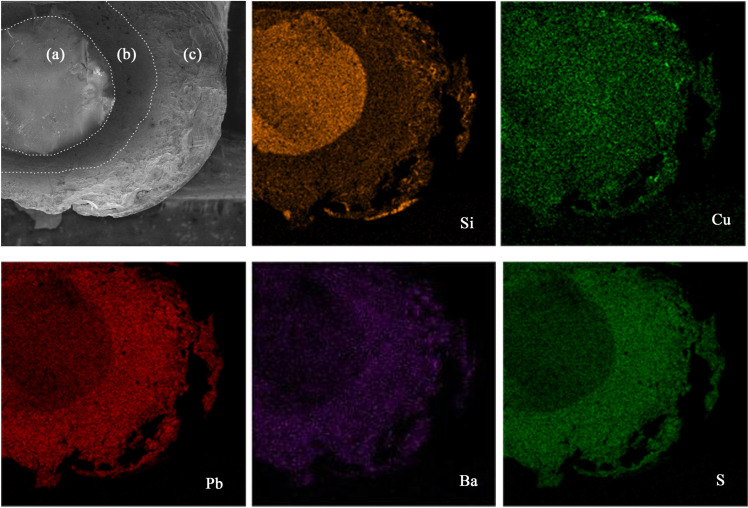
SEM and elemental mapping results of sample 2.

To further explore the weathering behavior of lead-barium glass, microscopic observation and elemental surface scanning of the cross-section of Sample 2 were carried out using SEM-EDS, the results are shown in Fig 5.

In the scanning electron microscope photos of the Sample 2 amplified by 100×, it can be observed that the microstructure of the sample substrate and the weathered layer (inner and outer) presents a very obvious difference, which can be divided into three layers: substrate (a), inner weathered layer (b), and outer weathered layer (c). The degree of weathering shows the characteristics of increasing in order from the inside to the outside. The substrate structure is smooth and dense, with melting bubbles of varying sizes visible. The inner weathered layer has a rough surface, but the structure is uniform and dense. The outer layer is loose and has a layered structure that diverges in the direction away from the glass substrate.

The elemental surface scanning results show that the content of Pb, Ba and S elements in the glass substrate is lower than that in the weathered layer. The content of Si element in the substrate is higher than that in the weathered layer, and the glassy degree is high at the center of the sample and decreases sequentially with the substrate outward. The content of Cu element in the substrate and the weathered layer is basically the same.

**Fig 6:**
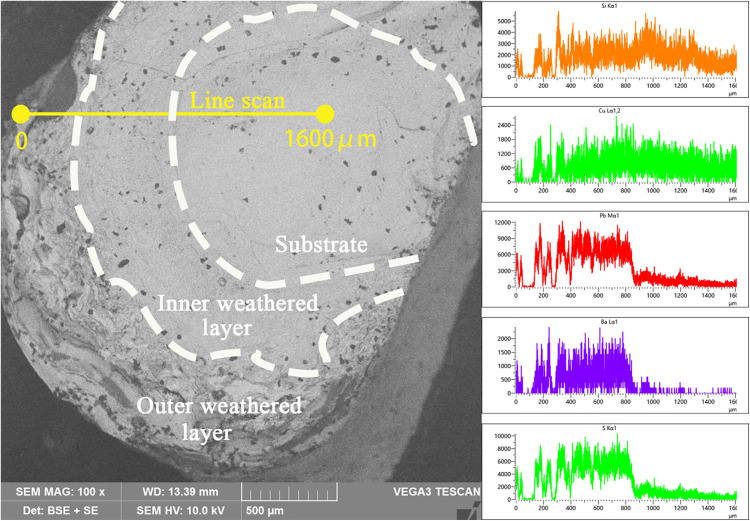
Backscatter image and line scan results of sample 2.

The study also observed the microscopic morphology of the cross section of Sample 2 in the backscattering mode, and a line scan was performed by selecting a line segment of about 1600 μm in length on the cross section according to the direction from the outside to the inside, as shown in Fig 6. Line scan results show that the outer weathered layer is mainly distributed in the range of 0-400 μm, the inner weathered layer in the range of 400-900μm, and the substrate in the range of 900-1600μm.The outer weathered layer has a loose structure with many voids, resulting in large fluctuations in elemental content and discontinuous analytical signals. The distribution of the elemental content of the inner weathered layer and the substrate is relatively uniform, which is consistent with the density of the microstructure.

The content of Pb, Ba and S elements in the substrate is obviously less than that in weathered layer. The content of Si element in substrate is slightly more than that in weathered layer. There is no significant change in the content of Cu element, which is consistent with the results of the surface scanning.

## Discussion

### Weathering mechanism of lead-barium glass

The materials of study in this paper are lead-barium glass samples, A suite of analytical techniques confirmed that the dominant weathering products are PbCO₃ and BaSO₄, a finding consistent with previous reports [25–29]. Elemental mapping across the cross-section of sample 2 reveals that, as alteration proceeds, Pb^2^⁺ and Ba^2^ ⁺ are progressively released from the glass substrate and are sequestered by CO₃^2^⁻ and SO₄^2^ ⁻ anions in the burial environment, precipitating as the sparingly soluble phases lead carbonate (PbCO₃) and barium sulfate (BaSO₄). Most of these precipitates remain in situ, leading to localized enrichment. This evidence demonstrates that the deterioration of lead–barium glass is driven not merely by physical disintegration of the network but by a chemically controlled reconstruction governed by ion migration and subsequent deposition.

The behaviour of Ba^2^⁺ during weathering is particularly complex. Raman spectroscopy conducted on successive weathered layers of sample 2 shows that BaSO₄ is relatively scarce in the newly formed layer adjacent to the pristine substrate, increases markedly within the intermediate (inner) layer, and diminishes slightly again in the outermost layer. Such a distribution can be attributed to the relatively high solubility of barium salts: Ba^2^ ⁺ readily enters pore waters as soluble species and is removed by percolating fluids, lowering its re-deposition efficiency near the surface. A similar trend was noted by Wang Yingzhu et al. (2023), who found Ba^2^⁺ to be more mobile than Pb^2^⁺ in Qin-period lead–barium glass fragments [4]; however, that study did not document element concentrations in the burial environment.

Building upon these earlier observations, the present work provides direct evidence for element migration within the burial milieu. Energy-dispersive X-ray spectroscopy (EDS) of soil residues adhering to the artefacts as shown in Table 2, the Pb contents of 0 to 0.91 wt % and Ba contents of 0.34 to 1.01 wt %. Notably, the Ba concentration consistently exceeds that of Pb, indicating a markedly greater mobility of Ba during weathering. This observation both corroborates prior findings and highlights a key risk factor for the long-term stability of buried lead–barium glass, preferential loss of Ba may render the weathered layer porous, ultimately compromising the artefact’s appearance and structural integrity.

**Table 2:** Results of energy spectrum analysis (wt%) of coated soil on sample surface.

Area	C	O	Na	S	Al	Si	P	Mg	K	Ca	Fe	Ba	Pb
1	20.52	50.49	0.71	1.01	5.03	14.94	/	/	1.75	1.89	3.00	0.34	0.33
2	15.78	51.26	0.71	1.10	5.44	16.28	0.08	0.01	1.88	3.43	3.42	0.32	0.29
3	7.27	53.25	0.64	1.50	6.39	19.32	0.11	0.14	2.35	4.00	4.30	0.74	/
4	8.44	47.98	/	1.63	7.14	18.38	0.07	0.06	2.80	5.91	5.67	1.01	0.91
Average	13.00	50.75	0.68	1.31	6.00	17.23	0.09	0.07	2.20	3.81	4.10	0.60	0.51

### Copper valence state transition

**Fig 7:**
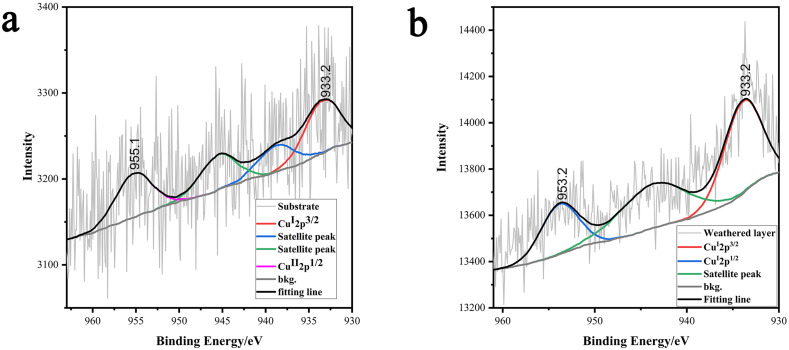
XPS fitting spectra of sample 2.

Studies have found that the Cu content in the glass remains largely unchanged before and after weathering, yet the color shifts from green to white. To investigate the changes in the oxidation state of Cu during the weathering process, X-ray photoelectron spectroscopy (XPS) was used to analyze the glass substrate and the weathered layer of sample 2. The analysis results are shown in Fig 7.

Peak fitting of the XPS results for the glass substrate shows that the peak at 933.2 eV corresponds to Cu(I) 2p3/2, and the peak at 955.1 eV corresponds to Cu(II) 2p1/2, indicating the presence of both Cu(II) and Cu(I) in the glass substrate [30]. By calculating the area ratio of the two peaks, the relative content of Cu(II) and Cu(I) is found to be 46% and 54%, respectively. In the XPS results for the glass weathering layer, the peaks at 933.2 eV and 953.2 eV correspond to Cu(I) 2p3/2 and Cu(I) 2p1/2, respectively, suggesting that the oxidation state of Cu in the weathering layer is exclusively Cu(I). This analysis reveals that the change in the valence state of copper is the fundamental cause of the color change in lead-barium glass.

Cu(Ⅱ) exhibits a sky-blue color in acidic glass and a green color in alkaline glass, while Cu(Ⅰ) is colorless and Cu(0) is red. When there is a significant amount of lead in the glass, Cu(Ⅱ) ions produce a bright green color in the glass substrate [31], which matches the color observed in the sample. This indicates that the atmosphere during the glass bead firing process was non-reductive, and the color in the glass substrate is primarily due to Cu(Ⅱ). As the glass substrate undergoes weathering, Cu(Ⅱ) is reduced to colorless Cu(Ⅰ). While Pb and Ba elements leach out, Cu(Ⅰ), due to its low solubility in water, remains in the substrate. During the weathering process, factors such as environmental temperature, humidity, pH, and surrounding pollutants contribute to the extraction and movement of metal ions, which attack and corrode the glass substrate, leaving behind weathered products that lack coloring ions.

## Conclusion

This study investigates three green lead-barium silicate glass beads excavated from Tomb M686 in the Qin Cemetery of Warring States Period in Hejia, Zhouling. To address the limited understanding of structural alteration and redox behaviour in earlier weathering studies, we employed a multi-analytical approach that includes SEM–EDS and XPS. The cross-section of Sample 2, in particular, allows a detailed visualization of ion migration and the stratified weathering zones.

Our results demonstrate that barium ions (Ba^2^⁺) are markedly more soluble than lead ions (Pb^2^⁺), a conclusion corroborated by elemental analyses of the burial soil surrounding the artefacts. XPS further reveals that the loss of the original green hue is primarily driven by the reduction of Cu(II) to Cu(I). These findings indicate that the weathering of lead–barium glass is controlled not only by selective ion migration but also by redox processes involving metallic species within the glass substrate.

This research represents the first integration of continuous SEM–EDS cross-sectional scanning with valence-state-resolved XPS for elucidating the weathering mechanisms of ancient Chinese lead–barium glass, surpassing previous studies that focused solely on surface alteration products. The outcomes deepen our understanding of ancient glass deterioration mechanisms and provide a scientific foundation for formulating targeted conservation strategies for glass artifacts.
